# Atypical case of neonatal-onset Gaucher disease type 3b: A case report

**DOI:** 10.1016/j.ymgmr.2025.101211

**Published:** 2025-03-29

**Authors:** Takanori Onuki, Kinuko Kojima, Kentaro Sawano, Nao Shibata, Yohei Ogawa, Go Hasegawa, Aya Narita, Hiromi Nyuzuki

**Affiliations:** a Department of Pediatrics, Niigata University Graduate School of Medical and Dental Science, 1-754 Asahimachi-dori, Chuo-ku, Niigata city, Niigata 951-8520, Japan; b Department of Pediatrics, Uonuma Institute of Community Medicine, Niigata University Medical and Dental Hospital, 4132 Urasa, Minamiuonuma city, Niigata 949-7302, Japan; c Department of Pediatrics, ISEIKAI International General Hospital, 4−14 Minami-Ogimachi, Kita-ku, Osaka city, Osaka 530-0052, Japan

**Keywords:** Gaucher disease, Gaucher disease type 3b, Gaucher disease type 1, Collodion skin, Neonatal onset, Pulmonary involvement, Lymphadenopathy

## Abstract

Neonatal-onset Gaucher disease (nGD) is considered perinatal lethal GD, a variant of GD type 2 (GD2), and is associated with collodion skin or hydrops fetalis, hepatosplenomegaly, and involvement of central nervous system (CNS). Pulmonary involvement (PI) and lymphadenopathy (LD) are reported GD complications and have unknown incidence, pathogenesis, and response to treatments. Here, we report the case of a patient diagnosed with nGD with collodion skin who developed only mild neurological symptoms and later died in early childhood due to treatment-resistant PI and LD. A female neonate was born at 38 weeks of gestation (weight: approximately 2012 g, height: 45 cm). She had a collodion skin, hepatosplenomegaly, hemorrhagic plaques, and cholestatic liver disease at birth. She was diagnosed with GD based on decreased glucocerebrosidase enzyme activity, and genetic analysis of *GBA1* revealed compound heterozygous mutations of c.1193G > T (p.Arg398Leu) and c.1265_1319del (p.Leu422fs). Intravenous enzyme replacement therapy (ERT) was initiated at the 15 days of age. At the age of 2 years and 2 months, she had a Developmental Quotient of 88 but developed horizontal gaze palsy. At 2 years 8 months of age, she developed mesenteric LD and PI because of which she failed to gain weight and developed tachypnea. She was started on oxygen therapy but died of respiratory failure and malnutrition due to PI and LD at the age of 3 years and 8 months. Pathological autopsy did not reveal the presence of Gaucher cells (GCs) in the liver, spleen, and bone marrow, but all lung macrophages had been transformed to GCs that were draining the alveoli, LD was observed in the mesenteric and mediastinal lymph nodes, and nodules of GCs were formed in bilateral kidneys. In conclusion, nGD with collodion skin is not always classified GD2. Although her phenotype may be classified as GD3b, her clinical course was like severe GD1. In addition, PI and LD are difficult to treat with adequate ERT.

## Highlights

• Our patient with Gaucher disease differed from previous reports on neonatal-onset.
• Neonatal-onset Gaucher disease with collodion skin is not always type 2.
• Pulmonary involvement and lymphadenopathy are difficult to treat with adequate ERT

## 1 Background

Gaucher disease (GD) is one of the lysosomal storage diseases. It is a rare, autosomal recessive genetic disorder caused by mutations in the *GBA1*(NM_001005741), which is located on chromosome 1q21 [1]. This leads to a deficiency of the lysosomal enzyme, glucocerebrosidase (GBA), which hydrolyzes glucosylceramide into ceramide and glucose [1]. GD is primarily categorized into three types based on clinical features related to involvement of central nervous system (CNS). GD type 1 (GD1, OMIM #230800) does not show involvement of CNS [1]. GD type 2 (GD2, OMIM #230900), called acute neuronopathic type and GD type 3 (GD3, OMIM #231000), called subacute neuronopathic type, are characterized by CNS involvement [1]. Patients with GD2 invariably exhibit clinical signs in the first year of life, although the type and severity of the manifestations vary widely [2]. Neonatal-onset GD (nGD) is considered perinatal lethal GD, a variant of GD2, is typically associated with hydrops fetalis or a collodion baby phenotype accompanied by congenital ichthyosis, hepatosplenomegaly, thrombocytopenia, anemia and failure to thrive due to progressive deterioration in the swallowing function [3]. This type is considered to have the poorest prognosis among all the GD types [4].

Pulmonary involvement (PI) and lymphadenopathy (LD) are reported GD complications [5]. PI falls into one of the following three categories: alveolar macrophage involvement, interstitial tissue involvement, and severe life-limiting pulmonary hypertension or hepatopulmonary syndrome [5]. LD refers to pathologically enlarged lymph nodes that may be localized or generalized. Mesenteric LD is associated with protein-losing enteropathy [5]. Although GD1 or GD3, severe GD genotypes such as p.Leu483Pro (previously known as Leu444Pro), and splenectomy are associated with the risk factors related to these complications [5], these incidence and natural history, pathogenesis, predictive factors, improvements and/or prevention of progression, and response to treatments such as intravenous enzyme replacement therapy (ERT) and substrate reduction therapy (SRT) are unknown. Furthermore, there are few reports of PI and LD in GD2, especially in nGD.

Here, we report the case of a patient diagnosed with nGD with collodion skin who developed only mild neurological symptoms and later died in early childhood due to treatment-resistant PI and LD.

## 2 Case report

**Fig. 1:**
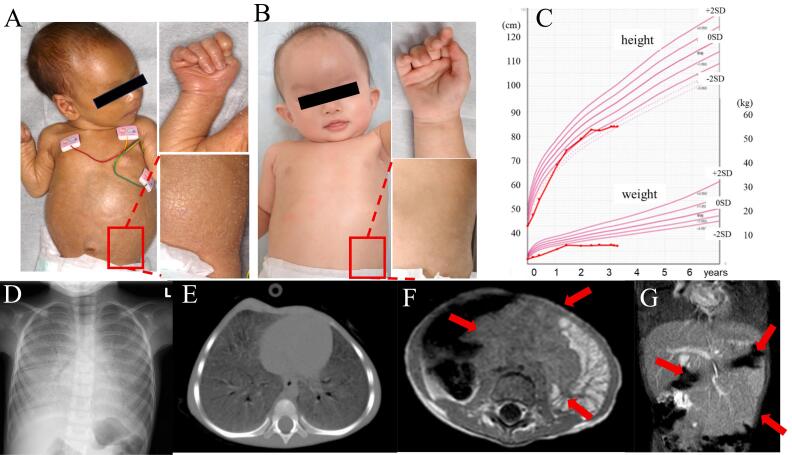
Physical and imaging findings of the patient. Photographs of the patient's external body at birth (Fig. A) and at nine months (Fig. B). She exhibited a collodion skin at birth. After enzyme replacement therapy, the collodion skin improved. Fig. C shows her growth curve. Decreased growth rate and poor weight gain was observed during two years after birth. Fig. D, E, F, and G show Chest X-ray, computed tomography (CT) image, and abdominal magnetic resonance imaging (MRI) image, respectively. These Figures demonstrate ground-glass opacity and enlarged mesenteric lymph nodes at the age of two years eight months.

A female neonate was born via elective C-section due to breech presentation at 38 weeks of gestation, weighted 2012 g (−2.2SD), heighted 45 cm (−1.5SD). Although her Apgar score was 8/8 (one minute /five minutes), she displayed a collodion skin, hepatosplenomegaly (palpable size of 4 cm), and hemorrhagic plaques at birth (Fig. 1A), and then she was admitted to the Neonatal Intensive Care Unit. Neurological findings were normal (hypotonia (−), Moro reflex (+), aspiration reflex (+), and grasping reflex (+)). Blood tests revealed low platelet count, cholestatic liver disease, coagulopathy, high ferritin level, elevated angiotensin converting enzyme (ACE), and TRACP-5b levels (Table 1). Computed tomography (CT) showed hepatosplenomegaly. GBA enzyme activity was low (0.23 pmol/h/disk; filtrate blood: reference value <3.0 pmol/h/disk, 1.4 nmol/h/mg; white blood cell: Mean 6.8 nmol/h/mg, Standard Deviation 1.9 nmol/ng/h, 1.2 nmol/mg protein/h; fibroblast: 0.9 % of normal control). *GBA1* (NM_000157.3) genetic analysis of her and her parents revealed compound heterozygous mutations of c.1193G > T (p.Arg398Leu) and c.1265_1319del (p.Leu422fs). Based on these results, the patient was diagnosed with GD. Due to thrombocytopenia and hepatosplenomegaly she received platelet transfusion and was being treated employing ERT with Imiglucerase (Cerezyme®, 60 U/kg/dose) since of 15 days of age. After initiating ERT, hepatosplenomegaly and the collodion skin improved (Fig. 1B); in addition, blood tests showed increased platelet counts, improved cholestatic liver disease, and decreased ACE levels. Interestingly, her growth and development progressed well and she did not have abnormal neurological symptoms including horizontal gaze palsy then. At the age of one year and four months, imiglucerase dose was increased from 60 to 120 U/kg/dose due to re-elevated ACE, AST, and ALT levels and residual hepatosplenomegaly. However, no improvement was observed. Anti-imiglucerase antibodies (IgG) were negative. Because the possibility of an attenuated effect of imiglucerase could not be denied, and the possibility of drug-induced liver injury was also suspected, ERT changed imiglucerase to velaglucerase alfa (VPRIV®, 120 U/kg/dose) at the age of one year and six months. As a result, AST and ALT levels and hepatosplenomegaly was improved. Despite the absence of dysphagia and gastrointestinal symptoms such as diarrhea, weight gain remained poor. The growth curve is shown in Fig. 1C. At the age of two years two months, the Developmental Quotient (DQ) using Kyoto Scale of Psychological Development 2001 [6] was normal (overall DQ 88, Postural-Motor 88, Cognitive-Adaptive 91, Language-Social 85; Low DQ is defined as that below 70). However, she developed horizontal gaze palsy. At the age of two years eight months, tachypnea and low SpO2 (96 % awake and 90 % asleep at room air) were observed. Chest radiography and CT revealed ground-glass opacity (Fig. 1D, E). Echocardiography did not indicate any evidences of pulmonary hypertension. At same time, her abdominal magnetic resonance imaging (MRI) revealed enlarged mesenteric lymph nodes which was not previously detected on abdominal echography (Fig. 1F, G). She developed PI and LD. Oxygenation was impaired because of PI and home oxygen therapy was initiated. However, her respiratory condition worsened, weight loss progressed, and she became progressively weaker. Because we determined that aggressive treatments with invasive such as a tracheostomy, respiratory support and intravenous nutrition therapy were not indicated based on disease conditions and her family consents, we provided palliative care treatment. She died of respiratory failure and malnutrition due to PI and LD at her age of three years eight months.

**Table 1:** Blood test at birth.

WBC	12,060	/μL	AST	108	U/L	Na	138	mEq/L	PT	10.1	%
Hb	11.7	g/dL	ALT	56	U/L	K	4.3	mEq/L	APTT	34.1	*sec*
Plt	83 × 10^3^	/μL	LDH	444	U/L	Cl	107	mEq/L	D-dimer	28.2	μg/mL
			ALP	1061	U/L	Fer	2799	ng/mL			
pH	7.42		T-Bil	9.3	mg/dL	Fe	99	μg/dL			
pCO2	28.5	mmHg	D-bil	6.1	mg/dL						
HCO3-	18.8	mmol/L	TP	6	g/dL	ACE	36.2	U/L			
BE	−5.5	mmol/L	BUN	10	mg/dL	TRACP5b	4880	mU/dL			
Lac	3.5	mmol/L	Cre	0.3	mg/dL						
			CK	1977	U/L						
			TBA	110.4	nmol/mL						
			NH3	81	μg/dL						

Reference value ACE 8.2–21.4 U/L, TRACP-5b 120–420 mU/dL, TBA <10.0 μmol/L.

ACE; angiotensin converting enzyme, TBA; total bile acid.

**Fig. 2:**
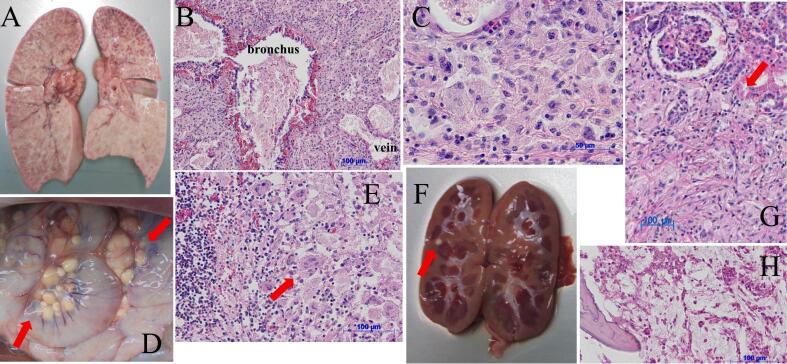
The findings of pathological autopsy. Fig. A shows gross pathological findings of the lungs. The white area is spread out in a speckled pattern, associating with Gaucher cells (GCs). Fig. B and C show microscopic findings of the lungs. Fig. C is a higher magnification image. An atypical massive of GCs are also observed. Fig. D and E show gross pathological and microscopic findings of the mesenteric and mediastinal lymph nodes, respectively. Numerous enlarged lymph nodes and massive involvement of GCs are observed (red arrow). Fig. F and G show gross pathological and microscopic findings of the kidneys. Nodules of GCs are observed in both kidneys (red arrows). Fig. H showed the microscopic findings of bone marrow. GCs were not present and bone marrow fat was absent.

Pathological autopsy revealed that Gaucher cells (GCs) were not present in the liver, spleen, and bone marrow (Fig. 2H); however, all lung macrophages had been transformed to GCs and were draining the alveoli (Fig. 2A, B and C); massive lymph nodes were observed throughout the whole body, especially in the mesenteric and mediastinal lymph nodes (Fig. 2D, E), and nodules of GCs were observed in the kidneys (Figure2F, 2G). Other findings included an absence of normal fatty tissue (subcutaneous fat, fat around the organs, and bone marrow fat) (Fig. 2H).

## 3 Discussion

Previous reports gave stated that nGD is classified as perinatal lethal GD, a variant of GD2, have the poorest prognosis among all the GD types in which a patient develops symptoms in the first year of life with CNS involvement [2,4]. Because the patient in our report displayed a collodion skin, hepatosplenomegaly, and thrombocytopenia at birth, we considered that she had perinatal lethal GD. However, she did not show only mild neurological symptoms such as horizontal gaze palsy thereafter. Nevertheless, later on, she developed PI and LD, which led to her death due to respiratory failure and malnutrition. Her clinical course differed from previous reports on nGD. Clinical types of GD3 are divided into three categories: GD3a, GD3b, and GD3c [7]. GD3b is mild phenotype characterized by gaze palsy only, whereas GD3a is more severe characterized by gaze palsy and various neurological symptoms [7]. GD3c is characterized by aortic and cardiac valve calcifications and hydrocephalus [7]. Although her phenotype may be classified as GD3b, her clinical course was like severe GD1. PI and LD are rarely reported complications of nGD or GD2. Patients with nGD may have died other causes before the onset of PI and LD because of its severe clinical manifestations. However, a report of autopsy cases of GD in Japan said that untreated neuronopathic type GD who died during childhood had PI (5/18) and LD (9/18) [8]. On the other hands, the absence of normal fatty tissues, such as subcutaneous fat, fat around organs, and bone marrow fat is atypical for malnutrition alone, and may be a factor related to some pathological manifestations.

To date, more than 500 *GBA1* mutations are associated with GD. However, predicting the phenotype or prognosis of genotype is difficult [7]. Although genotype-phenotype correlations in GD are often limited, some genotypes have an assumed phenotype. For example, the homozygous or compound heterozygous for *GBA1* mutation of p.Asn409Ser (previously known as Asn370Ser) is not associated with neuronopathic symptoms. In contrast, *GBA1* mutation of p.Leu483Pro (previously known as Leu444Pro) is most frequently associated with GD2 or GD3 [7]. The *GBA1* mutation seen in our patient was detected as compound heterozygous mutations of c.1193G > T (p.Arg398Leu) and c.1265_1319del (p.Leu422fs). Previously, *GBA1* mutation of c.1193G > T (p.Arg398Leu) was reported for GD2 [9,10] and that of c.1265_1319del (p.Leu422fs) is reported for GD1 [11]. Aries et al. [10] reported a female patient with GD2 whose *GBA1* was detected as the homozygous mutation of c.1193G > T (p.Arg398Leu); consequently, she was administered a high-dose ambroxol treatment, as per the pilot study for neuropathic GD as described by Narita et al. [12]. She had developed collodion skin at birth and was treated with ambroxol followed by ERT. Remarkably, she showed nearly age-appropriate cognitive and motor development at three years of age. Although ambroxol may have been effective for neurological symptoms in her case, *GBA1* mutation of c.1193G > T (p.Arg398Leu) may show a similar phenotype except PI and LD as our patient.

Current treatments for GD are mainly ERT, SRT, and supportive care. ERT and SRT have an effect on anemia, thrombocytopenia, hepatosplenomegaly, and skeletal improvement [13,14]. These treatments alone or in combination have been shown to be effective in some cases but have limited efficacy for the prevention and treatment of PI and LD [5,15,16]. Moreover, because recombinant enzyme does not cross the blood-brain barrier, there is no evidence that ERT and SRT reverse, stabilize, or slow the progression of neurological involvement [17]. Recently, it was reported patients with GD2 who were treated using ERT survived longer than those who were not, although all the neurological symptoms worsened as the disease progressed [18]. The patient in our report was treated using ERT alone. Since ERT displayed a dose-dependent improvement in hematological and visceral parameters in GD1 [19], the ERT dosage increased. Based on the findings of our patient's laboratory reports and pathologic autopsy, ERT treatment, regardless of dosage, was effective for hepatosplenomegaly, born marrow, and thrombocytopenia; however, it was ineffective for PI and LD. Therefore, developing novel strategies to treat such are desirable.

## 4 Conclusion

nGD with collodion skin was not always classified GD2 such as perinatal lethal GD. Although the patient's phenotype may be classified as GD3b because of horizontal gaze palsy, her clinical course was like severe GD1. PI and LD are difficult to treat with adequate ERT. Our case report was also valuable for future clarification of pathogenesis and correlation of phenotype-genotype in GD.

## Informed consent, and ethics of experimentation

This study was conducted in accordance with the guidelines of the Declaration of Helsinki. Permission to present the data in this report was obtained from the patients' parents.

## CRediT authorship contribution statement

**Takanori Onuki:** Writing – original draft, Investigation, Data curation. **Kinuko Kojima:** Investigation, Data curation. **Kentaro Sawano:** Investigation, Data curation. **Nao Shibata:** Data curation. **Yohei Ogawa:** Data curation. **Go Hasegawa:** Investigation, Data curation. **Aya Narita:** Supervision. **Hiromi Nyuzuki:** Writing – review & editing, Supervision, Investigation, Data curation.

## Declaration of competing interest

None.

## Data Availability

No data was used for the research described in the article.

## References

[1] Stirnemann J., Belmatoug N., Camou F. (2017). A review of Gaucher disease pathophysiology, clinical presentation and treatments. Int. J. Mol. Sci..

[2] Weiss K., Gonzalez A., Lopez G., Pedoeim L., Groden C., Sidransky E. (2015). The clinical management of type 2 Gaucher disease. Mol. Genet. Metab..

[3] Chida R., Shimura M., Ishida Y., Suganami Y., Yamanaka G. (2023). Perinatal lethal Gaucher disease: a case report and review of literature. Brain Dev..

[4] Grabowski G.A., Antommaria A.H.M., Kolodny E.H., Mistry P.K. (2021). Gaucher disease: basic and translational science needs for more complete therapy and management. Mol. Genet. Metab..

[5] Ramaswami U., Mengel E., Berrah A. (2021). Throwing a spotlight on under-recognized manifestations of Gaucher disease: pulmonary involvement, lymphadenopathy and Gaucheroma. Mol. Genet. Metab..

[6] Society for the Kyoto Scale of Psychological Development (2002).

[7] Daykin E.C., Ryan E., Sidransky E. (2021). Diagnosing neuronopathic Gaucher disease: new considerations and challenges in assigning Gaucher phenotypes. Mol. Genet. Metab..

[8] Hojo H. (1985). Pathological studies on the development and accumulation mechanisms of Gaucher cells. Nihon Mounaikeigakkai-Kaishi.

[9] Bulut F.D., Kör D., Şeker-Yılmaz B. (2018). Four Gaucher disease type II patients with three novel mutations: a single Centre experience from Turkey. Metab. Brain Dis..

[10] Aries C., Lohmöller B., Tiede S. (2022). Promising effect of high dose ambroxol treatment on neurocognition and motor development in a patient with neuropathic Gaucher disease 2. Front. Neurol..

[11] Erdos M., Hodanova K., Taskó S. (2007). Genetic and clinical features of patients with Gaucher disease in Hungary. Blood Cells Mol. Dis..

[12] Narita A., Shirai K., Itamura S. (2016). Ambroxol chaperone therapy for neuronopathic Gaucher disease: a pilot study. Ann. Clin. Transl. Neurol..

[13] Weinreb N.J., Goldblatt J., Villalobos J. (2013). Long-term clinical outcomes in type 1 Gaucher disease following 10 years of imiglucerase treatment [published correction appears in J Inherit Metab Dis. 2014 Jan;37(1):147]. J. Inherit. Metab. Dis..

[14] Cox T.M., Drelichman G., Cravo R. (2017). Eliglustat maintains long-term clinical stability in patients with Gaucher disease type 1 stabilized on enzyme therapy. Blood.

[15] Vellas D., Gramont B., Grange R., Cathébras P. (2021). Pulmonary involvement responsive to enzyme replacement therapy in an elderly patient with Gaucher disease. Eur. J. Case Rep. Intern. Med..

[16] Lee N.C., Chien Y.H., Wang C.H. (2022). Safety and efficacy of eliglustat combined to enzyme replacement therapy for lymphadenopathy in patients with Gaucher disease type 3. Mol. Genet. Metab. Rep..

[17] Vellodi A., Tylki-Szymanska A., Davies E.H. (2009). Management of neuronopathic Gaucher disease: revised recommendations. J. Inherit. Metab. Dis..

[18] Roshan Lal T., Seehra G.K., Steward A.M. (2020). The natural history of type 2 Gaucher disease in the 21st century: a retrospective study. Neurology.

[19] Grabowski G.A., Kacena K., Cole J.A. (2009). Dose-response relationships for enzyme replacement therapy with imiglucerase/alglucerase in patients with Gaucher disease type 1. Genet. Med..

